# Evidence and Case Report in a Portuguese Hospital: Is Therapeutic Ultrasound a Viable Solution in the Treatment of Mastitis?

**DOI:** 10.7759/cureus.67615

**Published:** 2024-08-23

**Authors:** Diogo Roxo, Brandon Allan, Sofia Campos, Raquel Branco, Mónica Bettencourt

**Affiliations:** 1 Physical Medicine and Rehabilitation, Hospital de Cascais, Lisbon, PRT; 2 Physical Medicine and Rehabilitation, Hospital do Divino Espírito Santo, Ponta Delgada, PRT

**Keywords:** breast pain, postpartum breast engorgement, mastitis, therapeutic ultrasound, breastfeeding

## Abstract

Mastitis is a very common maternal complication of lactation and contributes to early cessation of breastfeeding. Therapeutic ultrasound has been proposed as a non-invasive therapeutic alternative and has been added to the latest recommendations aimed at the treatment of mastitis spectrum disorders. In this article, we highlight the successful utilization of therapeutic ultrasound as part of the therapeutic program after the diagnosis of mastitis in a woman who was breastfeeding three newborn children.

## Introduction

Mastitis is an inflammation of the mammary glands that is usually distributed along their ducts, alveoli, and adjacent connective tissue [1]. It is a common maternal complication of lactation and contributes to early cessation of breastfeeding. In the past, mastitis has been regarded as a single pathological entity in the lactating breast. In fact, it affects up to 33% of women during lactation [2]. Scientific evidence now demonstrates that mastitis encompasses a spectrum of conditions that include ductal narrowing, area of thickening of the duct that makes breast tissue more tender; inflammatory mastitis, persistent or worsening ductal narrowing that presents with systemic symptoms such as fever and local inflammatory signs such as breast pain, erythema, and edema; bacterial mastitis, progression from inflammatory mastitis with worsening erythema and induration; and phlegmon and abscess, infected fluid collection [1].

Breastfeeding is recommended by the Centers for Disease Control and the World Health Organization for a period of at least six months [2]. It offers both infant and maternal benefits. This requires a moment of reciprocal learning between the mother and the infant that contributes to gaining a strong bond of affection between them both. Like all learning, it creates a space of vulnerability that translates into difficulties that arise during breastfeeding and that ultimately can lead to mastitis spectrum disorders [3]. Diseases in the mastitis spectrum, namely, bacterial or inflammatory mastitis, can lead to premature cessation of breastfeeding [2,4].

There are some risk factors that contribute to the occurrence of mastitis such as age, race, high socioeconomic position, the severity of functional loss, increased hospital length of stay, comorbidities (e.g., anemia, diabetes, drug abuse, smoking, hypertension, obesity and psychosis), primiparous women, cesarean delivery because of delayed skin-to-skin contact, dysbiosis, and hyperlactation [1,2,4,5]. The last two are described as the major risk factors for developing mastitis spectrum disorders. Dysbiosis is the disruption of the milk microbiome, and the risk factors include the regular use of breast pumps, the use of probiotics, exposure to antibiotics, cesarean births, and maternal genetics and medical conditions [1]. We must allow the exchange of microorganisms to occur between the baby's mouth and the mother’s breast, minimizing breast pump usage and the risk of dysbiosis. With regard to hyperlactation, the lactating breast works according to a positive feedback mechanism through the hormonal stimulation it receives. Thus, the greater the stimulus and number of feedings, the greater the milk production [1]. This positive feedback in milk production should make us not try to “empty” the breasts but rather feed the infant on demand.

Differential diagnosis should be made between mastitis and postpartum breast engorgement although, in reality, they share pathophysiological mechanisms. Postpartum breast engorgement occurs in 72%-82% of lactating women and is an inflammatory pathology that presents with bilateral breast swelling and pain with nipple-areolar complex edema, fever, malaise, and discomfort [1,3,6]. It usually occurs between the third and sixth days postpartum but can occur at any time during lactation [1,3,6]. Unlike what happens with mastitis, the symptoms of postpartum breast engorgement are self-limited and generally improve after 24 hours.

Although it is known that the pathophysiology associated with the occurrence of mastitis is complex and may involve the various risk factors previously explained, historically it is known that milk stasis leads to disruption of the lumen of the breast ducts [2]. The findings of the study conducted by Lavigne et al. suggest that two-thirds of breastfeeding women experience blocked ducts that can result in noninfectious mastitis [7]. A baby's poor latch to the breast, whether pathological (caused by anomalies in the baby's mouth) or behavioral (during the learning phase of latching on to the breast), contributes to mechanical irritation that can cause cracks and fissures and promote entry of bacteria. These lesions are risk factors for both mastitis and nipple infection [2]. This is why Staphylococcus aureus is the most common pathogen involved in bacterial mastitis [2,4,5]. Other pathogens include Streptococcus pyogenes and fungal species such as Candida albicans and Mycobacterium tuberculosis [2]. Taken as a common doubt in this context, household items and pumps should not be sterilized frequently because mastitis is not usually related to unhygienic practices [1]. Mastitis is not contagious and children should continue to be breastfed and consume milk from the infected breast as it is safe [1,4]. More than that, milk from a breast with mastitis has been shown to contain increased levels of some anti-inflammatory components that may be protective for the infant [8]. The exception is in women with human immunodeficiency virus, in whom breastfeeding should be discontinued as vertical transmission of this virus is more likely in the presence of mastitis [9,10]. It is possible that the baby does not like the taste of milk in the presence of mastitis due to the high sodium content [11]. In these cases, the milk should be pumped and discarded [11].

In line with what is done in the dairy industry, which uses the analysis of certain milk biomarkers, namely, somatic cell count (SCC), to determine the existence of inflammation of the mammary gland, in human milk a high sodium concentration and sodium/potassium ratio are markers of subclinical mastitis. A study published by Pace et al. concluded that the risk of developing clinical mastitis in the first six weeks postpartum is highest in women with an elevated SCC as early as the first week postpartum and, in addition, the risk of developing clinical mastitis is higher in women who had elevated SCC in milk in the previous week [12]. Still, these biomarkers have low accuracy in terms of prognosis in cases of clinical mastitis [12].

Regarding the recommendations and treatment of mastitis, we have spectrum-wide recommendations. These include reassuring mothers that many mastitis symptoms will resolve with conservative care and psychosocial support, feeding the infant on demand, minimizing breast pump usage, avoiding the use of nipple shields that will result in inadequate breast milk extraction, wearing an appropriately fitting supportive bra, not performing deep breast massage given the potential to increase tissue inflammation, avoiding cleaning of nipple due to the possibility of skin maceration and pain, avoiding unroofing associated nipple blebs, applying ice every hour or heat that may provide comfort for some patients, taking paracetamol 1,000 mg every eight hours and/or ibuprofen (maximum 800 mg every eight hours), taking soy lecithin or sunflower to take advantage of its anti-inflammatory potential, emulsifying milk, and finally applying a steroid cream to reduce inflammation on the surface of the nipple. In cases of bacterial mastitis, we should add antibiotic therapy. Empiric antibiotic therapy should be managed with dicloxacillin or flucloxacillin 500 mg, as the first line, four times daily for 10-14 days or with clindamycin 300 mg four times daily for 10-14 days. Furthermore, we should use targeted antibiotic therapy after milk culture data and antibiogram. It could be necessary for hospital admission and intravenous antibiotics if we have a known multidrug-resistant organism, severe sepsis, or inability to tolerate oral medication and fluid. In these cases, the mother must be hospitalized together with the baby to allow breastfeeding to continue on demand [1,4,5].

With regard to the utilization of therapeutic ultrasound, it has been proposed as a non-invasive alternative or adjuvant strategy to reduce inflammation and relieve edema [1,4]. Some studies suggest that therapeutic ultrasound can promote symptomatic improvement and be an important therapeutic adjuvant in mastitis spectrum disorders through its thermal (continuous therapeutic ultrasound) and non-thermal effects (pulsed therapeutic ultrasound). Randomized clinical studies have not yet been able to attest to the added value of using therapeutic ultrasound; however, it is actually an adjunctive treatment recommended by the Academy of Breastfeeding Medicine [1]. The effect of continuous ultrasound lies mainly in the application of heat, which can lead to the opening and dilation of the breast ducts, allowing them to unblock, and massage, which will reduce the tension of local soft tissue fibers, increase local blood flow that will nullify any anoxic stimulus present, increase the drainage of metabolic wastes, and stop the sensation of pain through the stimulation of local sensory nerve endings, taking advantage of the clinical benefits of both [3,6]. On the other hand, the effects of pulsed ultrasound depend on cavitation, acoustic streaming, and microstreaming [3]. The increase in cellular activity is, in this case, promoted by the ultrasound energy that is applied. In this way, a mechanical pressure wave is responsible for altering the properties of the cell membrane and creating microscopic bubbles in living tissues that will influence ion flows [3]. Increased cellular activity is the biological process responsible for the therapeutic effects of this ultrasound modality [13].

Cavitation is understood as the successive expansion and compression of gas-filled bubbles due to the effects induced by ultrasound on tissue fluids. A stable cavitation occurs when the size variation of the gas-filled bubbles does not reach a critical size. Within the therapeutic range of ultrasound (0,1-3 Watts per square centimeter or W/cm^2^) and applied to normal tissues, unstable cavitations are not expected to occur, except in air-filled cavities, such as lungs and intestines. Bubble growth can be managed according to ultrasound parameters. Therefore, a higher ultrasound frequency corresponds to a shorter cycle duration, and there is less time for bubbles to grow. The characteristic that distinguishes pulsed ultrasound in this aspect is the fact that it prevents continuous growth of the bubble, allowing it to return to its initial size during the off period [3].

Another phenomenon used by pulsed ultrasound devices is acoustic streaming. According to this phenomenon, the permeability of the cell membrane is modified through changes in sodium and calcium channels, allowing greater diffusion rates and changes in cellular secretions and protein synthesis. The initial modification of cell permeability is achieved by the presence of small-scale eddying of fluids near a vibrating structure [3].

The last of the physical phenomena of pulsed ultrasound is microstreaming. It is based on creating rotational and torsional movements in cells through the creation of currents in the layers of fluid that surround the vibrating bubbles [3].

In addition to therapeutic ultrasound, the use of other physical agents, such as acoustic pulse therapy (APT), also called shockwaves or radial waves, has been tested in the dairy industry to treat cows with mastitis. The use of antibiotics in dairy cows makes it impossible to collect any milk as it will be contaminated by antibiotic residues, which results in large financial losses for producers [14]. The study published by Leitner et al. [14] demonstrated that the use of APT allowed the treatment of cows with mastitis from very early stages, which allows to increase in the quantity and quality of the milk produced probably due to the increased healing process of the damaged tissues and to reduce the need to suspend milk production for a certain period. A higher cure of the bacteria, a faster return to the usual milking in clinical mastitis, and, finally, a decrease in culling of cows were also recorded [14]. To our knowledge, there are currently no published studies regarding the possible use of APT in humans for the treatment of mastitis spectrum disorders.

## Case presentation

We present a 25-year-old Brazilian woman, who had a cesarean delivery on June 29, 2022, from which three babies were born. She started soon breastfeeding the triplets, and on July 7, 2022, she was observed by an Obstetrician at Cascais Hospital due to inflammatory signs and exudate from the surgical wound and was medicated with ibuprofen 400 mg every eight hours. Four days later, the patient was reobserved due to severe breast pain, redness, and fever. She was medicated with amoxicillin + clavulanic acid (875+125 mg) every 12 hours for one week and maintained ibuprofen. In the next week, the patient was reassessed by Obstetrics of Cascais Hospital, maintaining fever and inflammatory signs in both breasts. She had induration of the breasts without fluctuation. Thus, was added clindamycin 150 mg every six hours to the previous anti-biotherapy, and she was referred to the Physical and Rehabilitation Medicine department.

**Figure 1:**
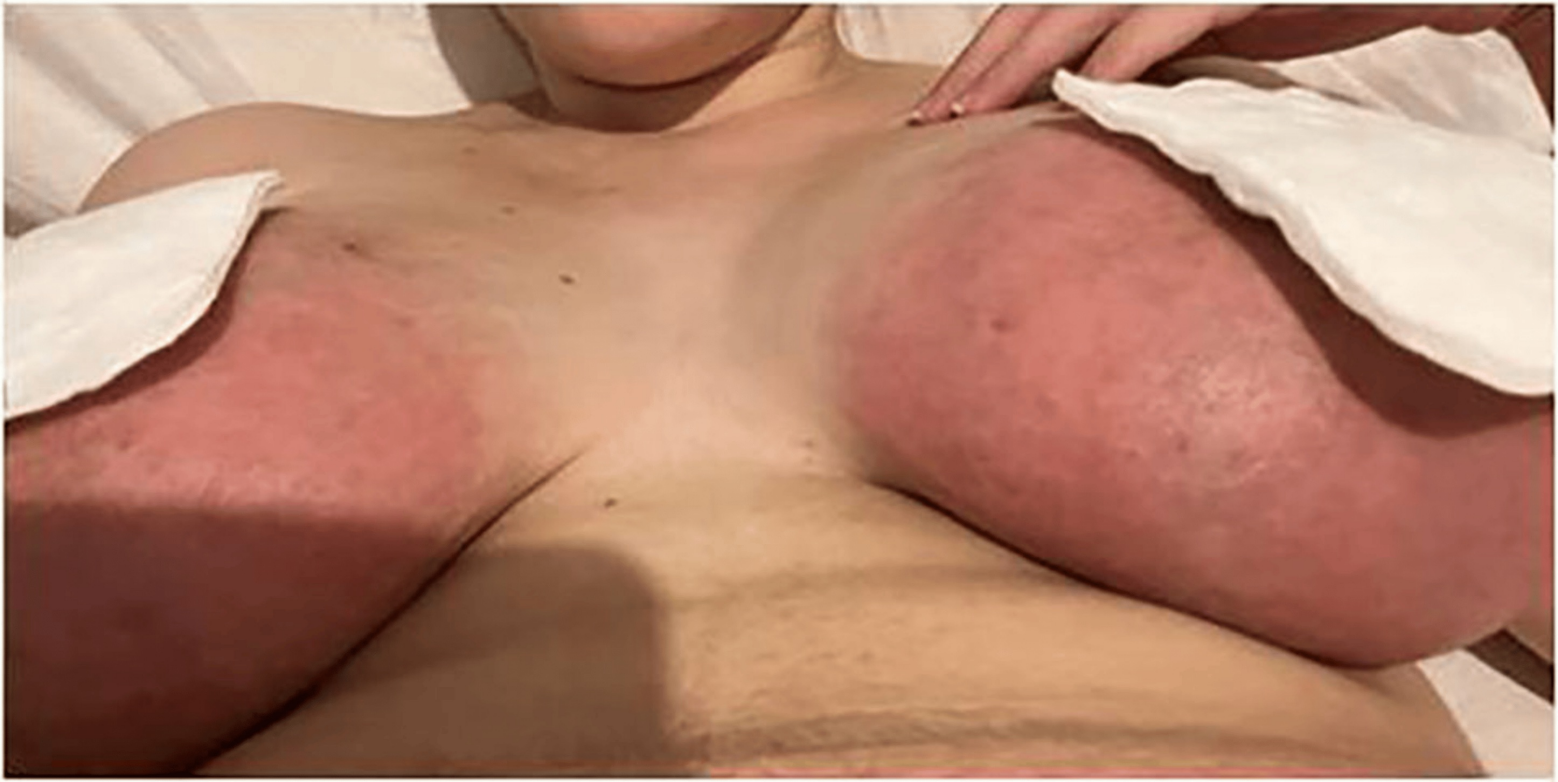
Day zero of continuous therapeutic ultrasound

On the next day, in the Physical and Rehabilitation Medicine appointment, the patient presented severe breast pain - eight out of ten according to the visual analogue scale (VAS) - and redness of both breasts (see Figure 1).

**Figure 2:**
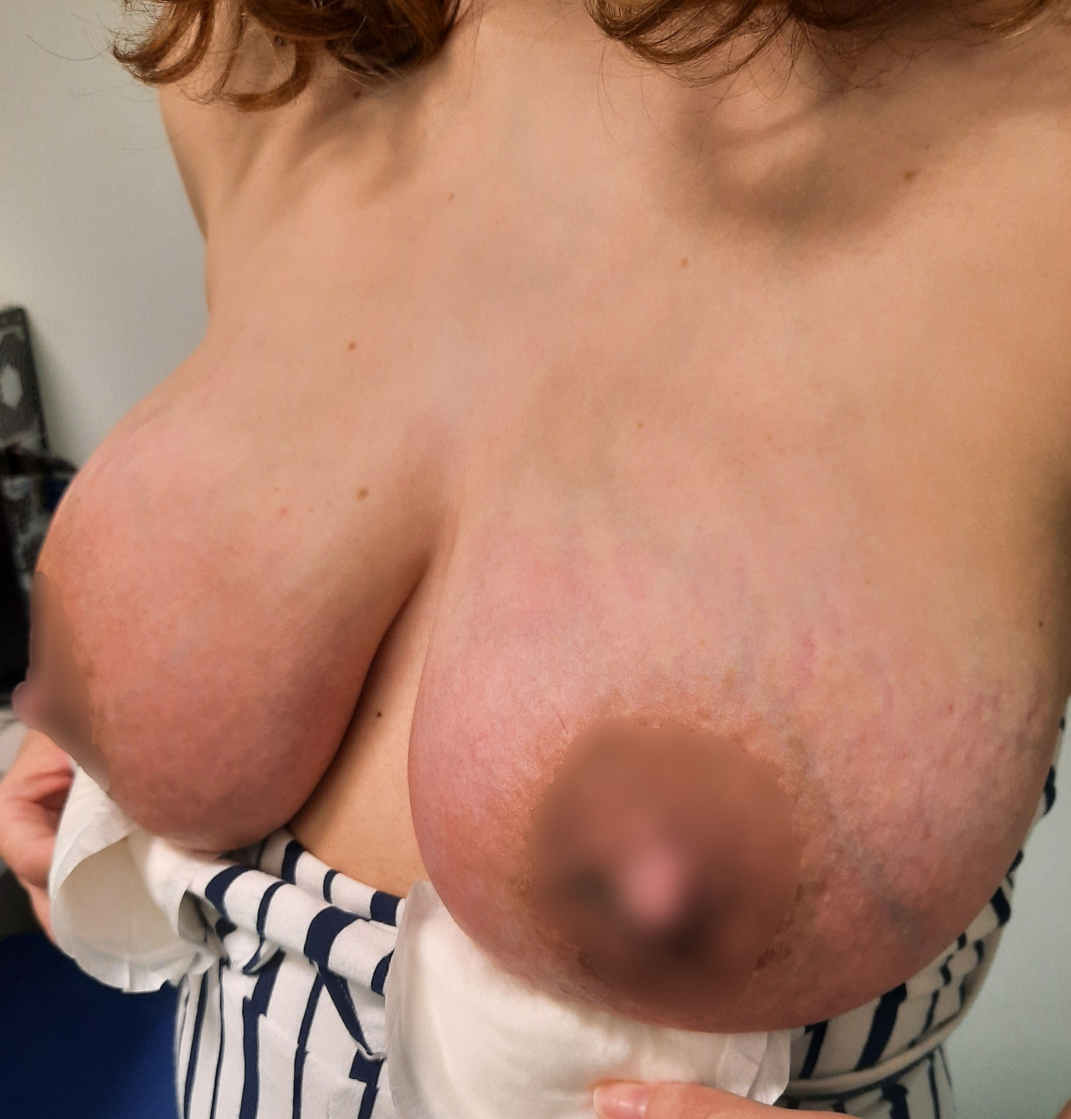
Day two of continuous therapeutic ultrasound

**Figure 3:**
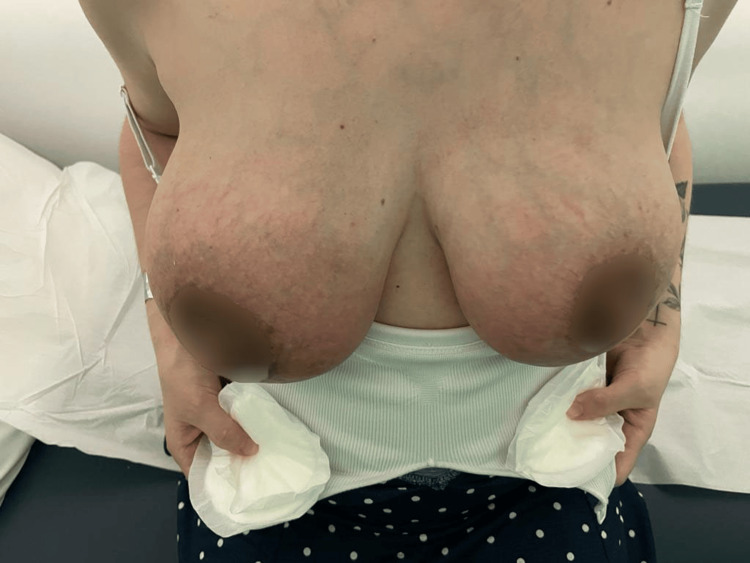
Day three of continuous therapeutic ultrasound

**Figure 4:**
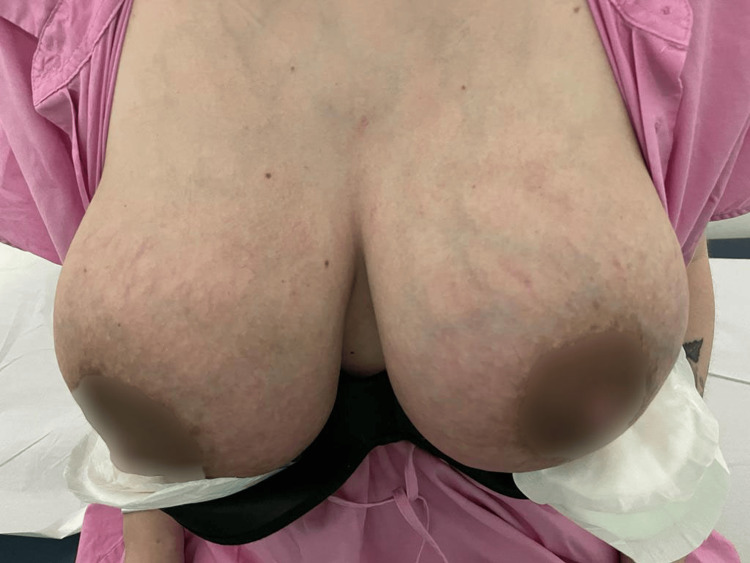
Day four of continuous therapeutic ultrasound

**Figure 5:**
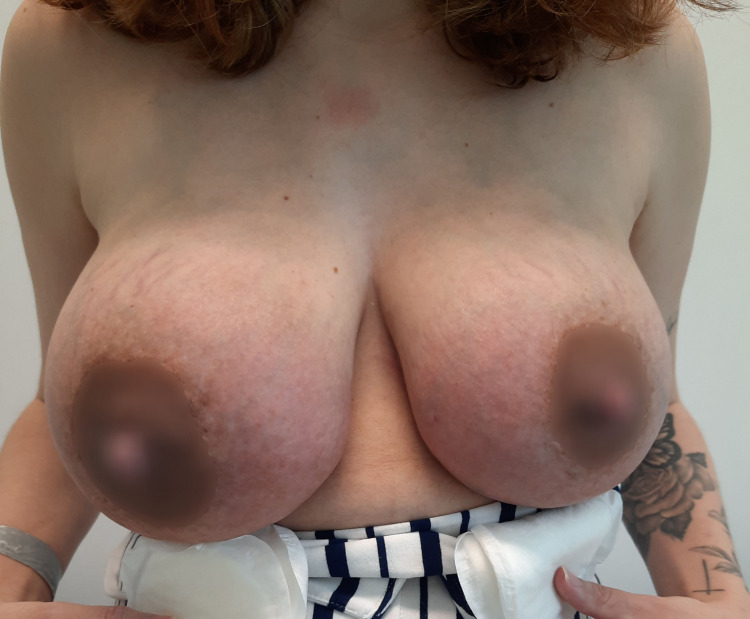
Day five of continuous therapeutic ultrasound

She was prescribed continuous therapeutic ultrasound with the following parameters: 1 megahertz (MHz) frequency, 2 W/cm^2^ intensity, and five minutes for each breast until clinical improvement. We completed one session per day for six days - see Figures 2-5.

After completing these sessions, the patient was reevaluated in a new Physical and Rehabilitation Medicine appointment, showing significant improvement in redness and breast pain - with two out of ten according to VAS - and was discharged. No adverse effects were reported. The patient did not undertake any other therapeutic strategy than the described drug therapy and continuous therapeutic ultrasound.

## Discussion

Treatment of mastitis includes the use of therapeutic ultrasound, although there is little scientific evidence. In 2022, the Academy of Breastfeeding Medicine promoted an update on recommended care in situations of mastitis and included the use of continuous therapeutic ultrasound with a moderate level of evidence and with a strength of recommendation level C. The recommended parameters are 1 MHz frequency, 2 W/cm^2^ intensity, and five minutes for each breast until clinical improvement [1]. In the clinical case presented, continuous therapeutic ultrasound was associated with the patient’s therapeutic regimen with the parameters suggested by the Academy of Breastfeeding Medicine, and an evident clinical improvement was obtained, which was quantified through the VAS. In addition to pain, there was a marked improvement in other inflammatory signs such as redness or edema, as can be seen in the photographic records provided.

Sankanagoudar et al. suggested, in a study involving 40 postpartum women, that the use of pulsed ultrasound accelerates symptomatic relief when compared to the exclusive use of other therapeutic strategies such as massage or manual expression of milk [3]. In another study involving 80 postpartum women, Priyanka et al. suggested that there is a significant clinical improvement when using pulsed ultrasound in addition to conventional treatment with heat and massage [6]. Regarding the use of continuous ultrasound, Lavigne et al. suggested that the majority of women reported resolution of symptoms with the application for eight to ten minutes with 1 MHz frequency and 2 W/cm^2^ intensity [7]. A study published by Shellshear et al. demonstrated that the application of continuous ultrasound prior to breastfeeding with parameters adjusted depending on whether the woman is primiparous or multiparous resulted in greater comfort for the woman during this moment and a subsequent reduction in breast tenderness [15]. On the other hand, an older study published by McLachlan et al. did not demonstrate a statistically significant difference in favor of the application of continuous ultrasound, which attributed the clinical improvement to factors unrelated to ultrasound, such as heat, massage, rest or the emotional and informational support given by health professionals [16]. Lin et al. concluded that the application of pulsed therapeutic ultrasound was not effective in improving breast symptoms in breastfeeding women, and therefore its inclusion in the treatment program should not be considered mandatory [17]. Other therapeutic methods have some scientific evidence regarding their effectiveness in women with breast engorgement, bringing them clinical benefits, such as Gua-Sha therapy [18] or the use of cabbage leaves [19].

## Conclusions

In mastitis, evidence on the utilization of therapeutic ultrasound is limited. Although in 2022 the Academy of Breastfeeding Medicine included therapeutic ultrasound in recommendations for the treatment of mastitis, it maintains a low level of evidence and strength of recommendation. This clinical case suggests that therapeutic ultrasound may be a successful strategy for managing mastitis. In this particular case, the patient was concomitantly medicated with antibiotic therapy; however, the introduction of therapeutic ultrasound seems to have been fundamental in the local resolution of the complaints and provided greater comfort to the patient during breastfeeding. More case series and randomized controlled trials are needed to confirm the potential benefit of therapeutic ultrasound in the treatment of mastitis.
